# Improper hydration induces global gene expression changes associated with renal development in infant mice

**DOI:** 10.1186/s12263-016-0544-0

**Published:** 2016-10-20

**Authors:** Chong-Su Kim, Dong-Mi Shin

**Affiliations:** 1 Department of Food and Nutrition, Seoul National University, Seoul, 08826 South Korea; 2 Research Institute of Human Ecology, Seoul National University, Seoul, 08826 South Korea

**Keywords:** Dehydration, Infancy, Kidney transcriptome, Renal development

## Abstract

**Background:**

The kidney is a major organ in which fluid balance and waste excretion is regulated. For the kidney to achieve maturity with functions, normal renal developmental processes need to occur. Comprehensive genetic programs underlying renal development during the prenatal period have been widely studied. However, postnatal renal development, from infancy to the juvenile period, has not been studied yet. Here, we investigated whether structural and functional kidney development was still ongoing in early life by analyzing the renal transcriptional networks of infant (4 weeks old) and juvenile (7 weeks old) mice. We further examined the effects of dehydration on kidney development to unravel the mechanistic bases underlying deteriorative impact of pediatric dehydration on renal development.

**Methods:**

3-week-old infant mice that just finished weaning period were provided limited access to a water for fifteen minutes per day for one week (RES 1W) and four weeks (RES 4W) to induce dehydration while control group consumed water ad libitum with free access to the water bottle. Transcriptome analysis was conducted to understand physiological changes during postnatal renal development and dehydration.

**Results:**

Kidneys in 4-week- and 7-week-old mice showed significantly distinctive functional gene networks. Gene sets related to cell cycle regulators, fetal kidney patterning molecules, and immature basement membrane integrity were upregulated in infantile kidneys while heightened expressions of genes associated with ion transport and drug metabolism were observed in juvenile kidneys. Dehydration during infancy suppressed renal growth by interrupting the SHH signaling pathway, which targets cell cycle regulators. Importantly, it is likely that disruption of the developmental program ultimately led to a decline in gene expression associated with basement membrane integrity.

**Conclusions:**

Altogether, we demonstrate transcriptional events during renal development in infancy and show that the impacts of inadequate water intake in the early postnatal state heavily rely on the impairment of normal renal development. Here, we provide a meaningful perspective of renal development in infancy with a molecular and physiological explanation of why infants are more vulnerable to dehydration than adults. These results provide new insights into the molecular effects of dehydration on renal physiology and indicate that optimal nutritional interventions are necessary for pediatric renal development.

**Electronic supplementary material:**

The online version of this article (doi:10.1186/s12263-016-0544-0) contains supplementary material, which is available to authorized users.

## Background

The kidney is a major organ that plays crucial roles in homeostasis in the body. It filters the blood to excrete waste products from the body [17] and controls body fluid volume, solute, and acid balance within a tight range in cooperation with other endocrine organs [33]. The critical functions of the kidney in the fluid regulatory system are implicated in precise regulation of plasma osmolality. When the body senses changes in osmolality, osmoreceptors at the hypothalamus stimulate arginine vasopressin (AVP) release, which promotes fluid reabsorption in the kidney and thirst by which water retention increases [30].

Kidney organogenesis comprises several common developmental steps: proliferation and differentiation of stem cells, epithelial-mesenchymal cell transition, branching, and regional segmentation [13, 16, 46]. Complex gene regulatory networks mediate cell interactions underlying anatomical and functional modifications in kidney morphogenesis [23, 45]. Especially, dynamic regulations underlying cell and tissue integrity are essential in kidney function and are highly associated with renal disorders [49]. A highly selective permeable glomerular barrier membrane establishes networks with podocyte foot processes and slit diaphragm, a modified form of adherens junction, to gain mature filtration functions [36]. Thus, retaining a stable cell and junction architecture is required for normal renal function [26, 36, 49].

Body water constitutes cells and tissues and functions as a carrier of nutrients and excretions [17, 29]. It also serves as both reactant and product in metabolisms [17]. Optimal hydration is necessary for a healthy life [9]. It is well documented that dehydration negatively impacts multiple physiological disorders [9, 17, 33]. Healthy adults regulate fluid balance to maintain homeostasis whereas infants have an immature fluid regulatory system [37] with a higher body water composition, reaching 75 % [9]. Moreover, infants, right after weaning, are more prone to drink insufficient water since caregivers are likely to be unaware of their thirst [9]. In this respect, infants seem to be at higher risk for dehydration.

While recent studies have shown that further growth of the nephrons and elongation of tubular structures occur after birth [8], little is known about how genetic events are regulated in renal growth postnatally [2, 7, 24, 50]. Moreover, the molecular and physiological background for why infants are more vulnerable to dehydration has not been studied yet, and there are few studies on whether dehydration causes physiological changes in infants. We hypothesized that fundamental mechanisms of pediatric dehydration may underlie renal developmental processes. To confirm this assumption, we studied the effects of insufficient water intake in infant mice, with emphasis on physiological and transcriptional changes in the developing kidney.

In this study, we performed global gene expression profiling analysis and identified the functional networks of differentially expressed genes in the kidneys of infant and juvenile mice. We showed that kidneys undergo biological processes to achieve structural and functional maturation even after birth and dehydration altered gene networks associated with normal renal development and maturation. These results may provide evidence that sufficient water intake from infancy to juvenile period is necessary for normal renal physiology and system development.

## Methods

### Experimental animals

Right after weaning, 3-week-old male C57BL/6 mice were housed for 1 and 4 weeks and sacrificed at the age of 4 and 7 weeks old, corresponding to infant and juvenile. Animals were randomly assigned into control (CON) and water restriction group (RES). A total of four groups were used in comparison analysis: CON 1W (4 weeks old, *n* = 5), CON 4W (7 weeks old, *n* = 6), RES 1W (4 weeks old, *n* = 6), and RES 4W (7 weeks old, *n* = 8). Animals were maintained in a 12-h light/dark cycle and fed ad libitum with an AIN-93G diet. Animals from water restriction group were maintained dehydrated until they were sacrificed, 24 h after the last provision of water bottle. All experimental procedures were approved by the IACUC (Institutional Animal Care and Use Committee) of Seoul National University and conducted according to the IACUC guidelines.

### Water restriction

Animals in water restriction group were imposed limited access to water. A water bottle was given to animals for 15 min a day during the experimental period. Control mice consumed water ad libitum with free access to the water bottle.

### Plasma biochemical analysis

Serum biochemical analysis for blood urea nitrogen (BUN) and serum creatinine (sCr) was conducted using a dry-chemistry blood analyzer, Spotchem SP-4410 (Arklay, Kyoto, Japan). Plasma osmolality was determined by Fiske 210 Micro-Sample Osmometer (Fiske, Norwood, MA, USA).

### RNA isolation

Total RNA was extracted from the kidneys and brain using DNA-free RNA isolation kit (RNAqueous-4PCR kit; Ambion, Austin, TX) according to the manufacturer’s directions. Total RNA integrity and quantity were assessed with a NanoDrop 2000 Spectrophotometer (Thermo Fisher Scientific, Wilmington, DE).

### Microarray hybridization

RNA samples were amplified for microarray analyses using Illumina TotalPrep RNA Amplification Kit (Ambion, Austin, TX). Five hundred nanograms of total RNA was used to prepare labelled complementary RNA (cRNA) with overnight incubation. Amplified cRNA was hybridized on Illumina MouseWG-6 Expression BeadChip arrays. The arrays were scanned with BeadArray Reader (BeadStation 500G Instrument, Illumina Inc.). Identification and quantification of spot images were obtained by Genome Studio software v1.0.2 (Illumina Inc.).

### Bioinformatic analysis of microarray data

The analysis was performed as described in [41]. Raw data was log-transformed and normalized by quantile normalization using Genome Studio software (Illumina Inc.). Differentially expressed genes among four groups were identified using an ANOVA (*p* < 0.05) by Partek® Genomics Suite software v6.6 (Partek, St Louis, MI) (http://www.partek.com/partekgs). Average expression levels of genes were compared between groups, and calculated *p* values were corrected for multiple comparisons using false discovery rate algorithm. Significant genes with fold change >1.5 and FDR <0.01 were used for further analysis.

Hierarchical clustering analysis was performed using the Pearson correlation distance matrix with average linkage algorithm in Genesis software v1.7.5 [43]. Functional categories of significant genes were determined by a right-tailed Fisher exact test. To examine the significance of functional categories which were classified based on Ingenuity Knowledge Base, Gene Set Enrichment Analysis was carried out (http://www.broadinstitute.org/gsea/index.jsp). Mechanistic networks underlying signaling pathways and metabolic pathways were built based on the Ingenuity Knowledge Base. Upstream regulator analysis was carried out to predict upstream regulators in direct or indirect relationships with dataset using Ingenuity Pathway Analysis (IPA) [22]. Functional network map of gene sets was constructed by using CytoScape software v3.2.0 (http://cytoscape.org) [40]. The microarray dataset is available at the Gene Expression Omnibus (www.ncbi.nlm.nih.gov/geo/, accession number GSE75604).

### Quantitative RT-PCR

DNase I treated total RNA was converted into cDNA by two-step procedure with MessageSensor RT kit (Ambion, Austin, TX), and messenger RNA (mRNA) levels were quantified by SYBR-GREEN qPCR method (Applied Biosystem, Carlsbad, CA). Relative mRNA expression level was calculated by ΔΔC_*T*_ method. Glyceraldehyde-3-phosphate dehydrogenase (GAPDH) was used as a housekeeping gene for normalization of mRNA expression of each sample. Primer sequences are as follows: *Avp*: forward 5′-CCAGGATGCTCAACACTACG-3′, reverse 5′-CTCTTGGGCAGTTCTGGAAG-3′; *Ccnd1*: forward 5′-CGTGGCCTCTAAGATGAAGG-3′, reverse 5′-CTGGCATTTTGGAGAGGAAG-3′; *Cdkn2b*: forward 5′-AGATCCCAACGCCCTGAAC-3′, reverse 5′-CGCAGTTGGGTTCTGCTC-3′; and *Gapdh*: forward 5′-TGCACCACCAACTGCTTAG-3′, reverse 5′-GATGCAGGGATGATGTTC-3′.

### Western blot analysis

Proteins were extracted from the kidneys. Equal amounts of protein were subjected to SDS-PAGE and transferred to PVDF membranes. The membranes were incubated with anti-SMO (1:10000, ab72130) and anti-GLIS1 (1:1000, ab105873) primary antibodies from Abcam (Abcam Inc., Cambridge, MA). Anti-α-tubulin (1:10000, T5168) from Sigma (Sigma Chemical Co., St. Louis, MO) was used as control. Horseradish peroxidase-conjugated goat anti-rabbit secondary antibody (1:5000, #7074S) from Cell Signaling Technology (Cell Signaling Technology Inc., Danvers, MA) and goat anti-mouse secondary antibody (1:10000, G21040) from Invitrogen (Invitrogen, Carlsbad, CA) were used for detection. Protein bands were visualized using ECL Western blot detection reagents (Amersham Pharmacia Biotech, Piscataway, NJ).

### Statistical analysis

Data are expressed as mean ± SEM. Statistical significance (*p* < 0.05) was evaluated by unpaired Student’s *t* test between two groups. Statistical analyses were performed using GraphPad Prism 6 software (GraphPad Software Inc., La Jolla, CA).

## Results

### Time-limited access to water bottle induces mild dehydrating physiological conditions in infant mice

**Fig. 1:**
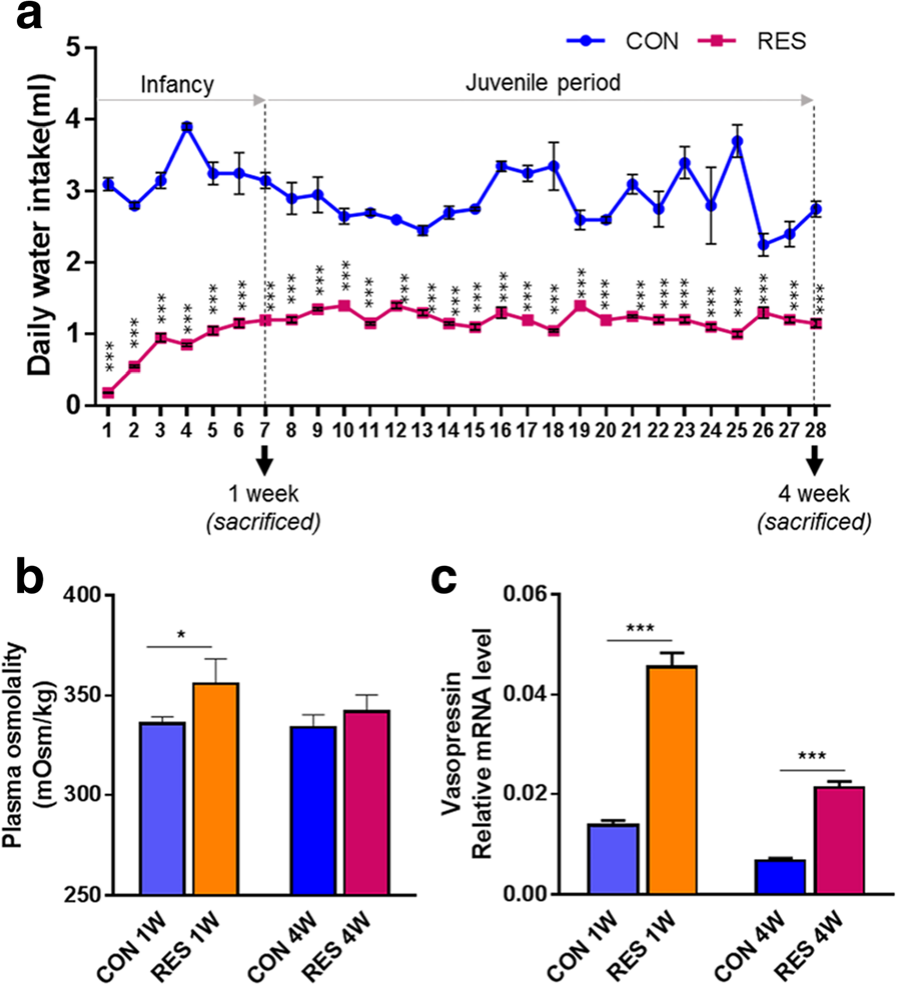
Mild dehydration was induced in infant mice. To observe the effects of prolonged dehydration on development, an experiment was performed from infancy to the juvenile period. The restriction group had limited access to the water bottle for 15 min a day. The water restriction group had reduced daily water intake compared to the control group (**a**). Mild dehydration was generated, resulting in elevated plasma osmolality (**b**) and brain vasopressin mRNA level (**c**) in the dehydrated mice. The number of samples in each group is as follows: CON 1W (*n* = 4), RES 1W (*n* = 4), CON 4W (*n* = 3), and RES 4W (*n* = 4). Data are expressed as mean ± SEM. Student’s *t* test; **p < 0.01, ***p < 0.001 versus control group

Infant mice that just finished weaning were provided limited access to a water bottle for 15 min per day for 1 week (RES 1W) and 4 weeks (RES 4W). Mice in the RES 1W group consumed 2.4 times less water compared to the age-matched control mice in the CON 1W group with ad libitum water intake (*p* < 0.001) (Fig. 1a). Mice in the RES 4W that grew into juveniles with the same daily water-restriction treatment showed a significant reduction in daily water intake, on average down to a third of that in control mice (CON 4W) (*p* < 0.001). In order to verify whether the reduction in water intake was sufficient to induce physiological changes in those mice, we determined the levels of plasma osmolality. Mice in RES 1W had significantly higher plasma osmolality than mice in CON 1W (*p* < 0.001). The 4-week dehydration resulted in increased plasma osmolality as well, although it was not significant (*p* = 0.083) (Fig. 1b). Next, we measured the transcript level of vasopressin in the brain and found dramatic increases in vasopressin production in both RES 1W and RES 4W, compared to CON 1W and CON 4W, respectively (*p* < 0.001) (Fig. 1c).

### Transcriptome profiling analysis of the kidneys in dehydrated mice

**Fig. 2:**
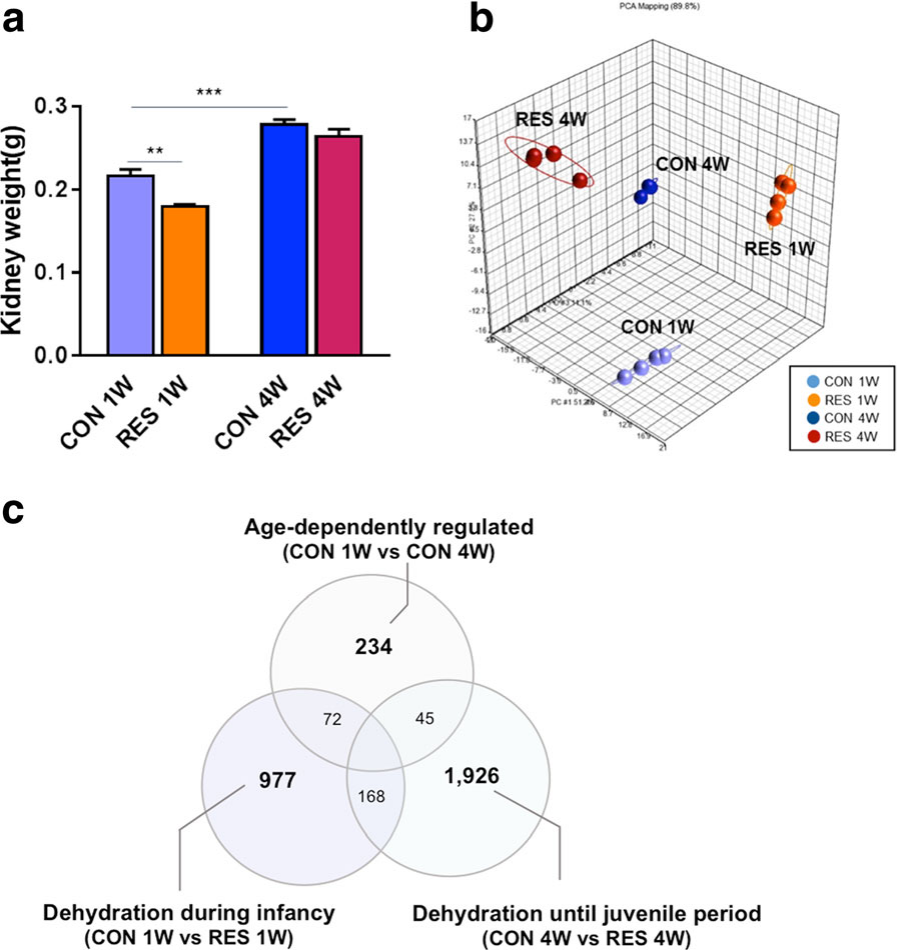
Transcriptome profiling analysis of the kidneys in dehydrated and control mice using genome-scale Illumina microarray. **a** The weight of the kidney increased with age, but dehydration caused decreased weight in the kidneys of infant mice (RES 1W) by 22 % and in juvenile mice (RES 4W) by 8 %. **b** A 3D principal component analysis (PCA) plot of the renal transcriptome data represents significantly distinguishable profiles for each group: CON 1W (*light blue*), RES 1W (*orange*), CON 4W (*blue*), and RES 4W (*red*). *Each dot* indicates an individual kidney sample. **c** The Venn diagram shows the number of probes that were detected with different signal intensity in each dataset. Data are expressed as mean ± SEM. Student’s *t* test; **p < 0.01, ***p < 0.001 versus control group

To examine the effect of dehydration in the developing mouse, the organ weight was measured. The weight of the kidneys increased with age from 0.22 g in CON 1W to 0.28 g in CON 4W (*p* < 0.001); however, dehydration caused a decrease of kidney weight in infant mice (RES 1W) by 22 % (*p* < 0.01) and juvenile mice (RES 4W) by 8 % (*p* = 0.23) compared to age-matched controls (Fig. 2a). To understand the molecular events underlying kidney growth and development from the age of infancy to the juvenile stage during the life cycle and to unveil the mechanism by which the kidneys’ weight were reduced while developing mice were under dehydration status, we conducted gene expression profiling analysis of kidneys using a genome-scale microarray.

Principal component analysis (PCA) showed that the transcriptome profiles were readily distinguished by either age (CON 1W and CON 4W) or a period of sustained water restriction (RES 1W and RES 4W) (Fig. 2b). Differentially expressed genes among the four groups were identified by ANOVA analysis (*p* < 0.05). A total of 234 probes were differentially expressed depending on age in the normal control groups (CON 4W vs. CON 1W). Furthermore, 977 probes were detected to distinctively mediate the transcriptional events in the infantile kidneys of the dehydration group (RES 1W vs. CON 1W), while 1926 differentially expressed probes were in the juvenile kidneys of the dehydration group (RES 4W vs. CON 4W) (Fig. 2c).

### Normal infant and juvenile mice present distinct renal transcriptional profiles

**Fig. 3:**
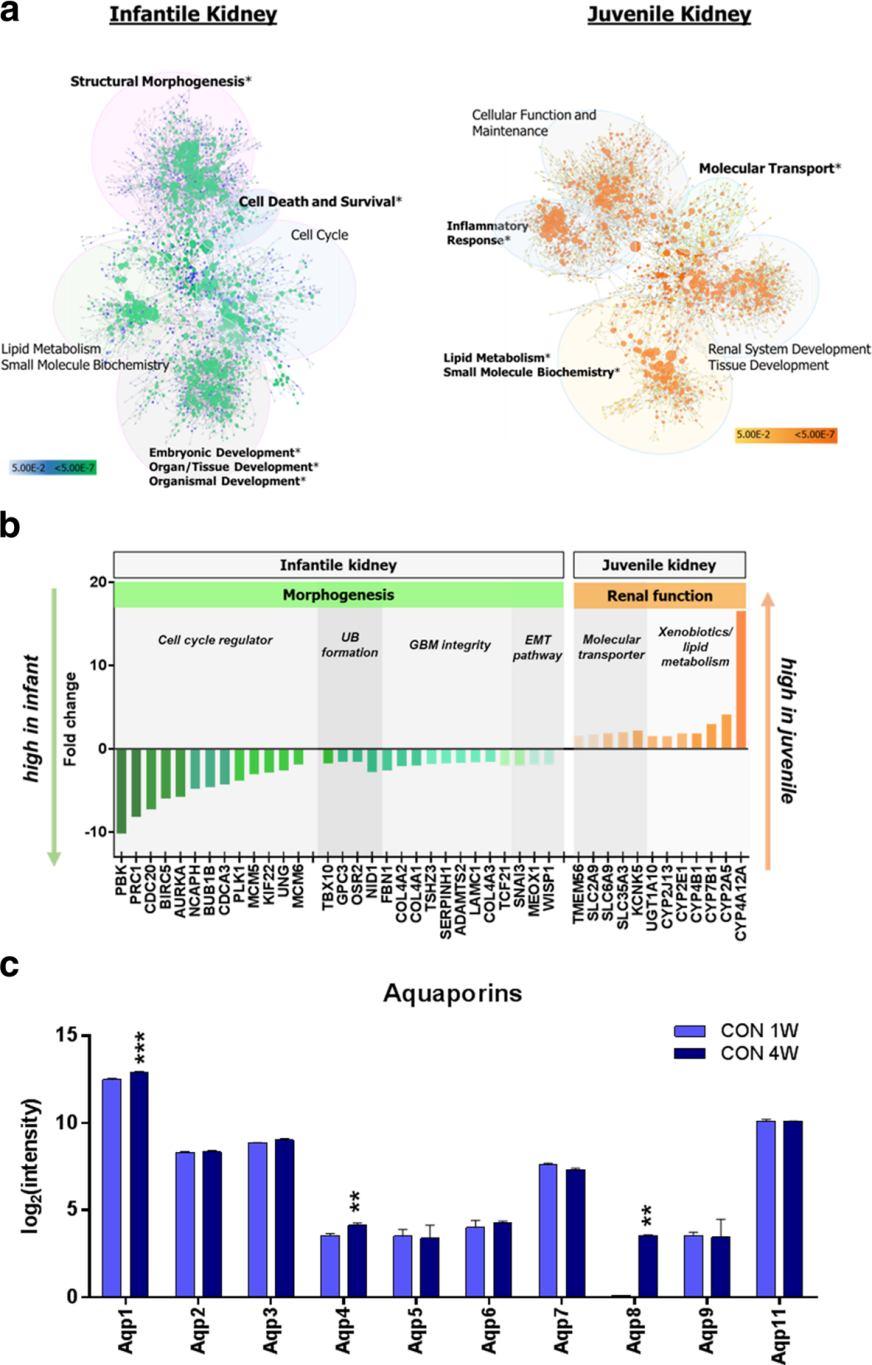
Distinctive functional network of genes in normal renal development during postnatal life. **a** Functional network of gene sets of CON 1W (4 weeks old; infant) and CON 4W (7 weeks old; juvenile). Gene ontology (GO) was categorized based on Ingenuity Pathway Analysis (IPA) knowledge. GO terms in *bold* with *asterisk* indicate enriched biological functions in gene set enrichment analysis. *Each node* represents a biological function and its color and size indicate the *p* value and number of genes in the cluster, respectively. **b** Identification of significant genes related to postnatal renal development. **c** The increasing transcript levels of aquaporin family with age support that functional maturation were taking place in the kidneys of infant and juvenile mice. *UB* ureteric bud, *GBM* glomerular basement membrane, *EMT* epithelial-mesenchymal transition. Data are expressed as mean ± SEM. Student’s *t* test; *p < 0.05, **p < 0.01, ***p < 0.001

Hierarchical clustering analysis showed that postnatal kidneys at different time points (CON 1W and CON 4W) had distinguishable transcriptome profiles (Fig. 3a). Two hundred thirty-four probes were differentially expressed in a comparison of CON 1W and CON 4W, with 149 probes upregulated in the kidneys of infant mice (CON 1W) and 85 probes upregulated in juvenile mice (CON 4W): gene list is given in Additional file 1. This finding implies that the kidneys of infant and juvenile mice might undergo different biological processes.

To extend our knowledge about the biological process occurring in the infantile kidney, we established a functional network map of differentially expressed genes and assigned gene ontology (GO) terms. Enriched GO categories from gene set enrichment analysis revealed that genes related to the cell cycle, tissue morphology, and development were significantly upregulated, whereas they were downregulated in CON 4W (Fig. 3a). Genes associated with cell cycle, which was the most significant biological process in the infantile kidneys, including *Aurka*, *Birc5*, *Bub1b*, *Cdc20*, *Meox1*, *Pbk*, *Kif22*, *Mcm5*, *Mcm6*, *Ncaph*, *Plk1*, *Ung*, and *Cdca3* were overexpressed (Fig. 3b), indicating that developmental cellular processes were still abundant in the kidneys of CON 1W. Among those genes, *Aurka*, *Plk1*, *Mcm5*, and *Kif22* have been found to function in embryonic renal development by mediating cell division [21]. In addition, *Birc5*, which had about a sixfold increase in infant mice, has been reported to be expressed only in the tubules and glomeruli of fetal kidney [39]. *Meox*, with a twofold increase in CON 1W compared to CON 4W, is one of the important factors in kidney formation and has a role in epithelial-mesenchymal cell interactions [34]. Genes that are essential for DNA synthesis and cell division (*Nasp*, *Rrm1*, *Rrm2*, *Prc1*) were upregulated and then eventually downregulated with age in our dataset. Many of the predictive upstream regulators within gene sets of CON 1W were associated with cell cycle regulation, including *Myc*, *Tp53*, *Foxm1*, *Ccnd1*, and *Smad3* (Additional file 2). *Myc* is highly expressed in the metanephros during renal organogenesis, and its disruption is associated with reduced cell proliferation [1]. These results imply that pathways mediating cell cycle progression were dominantly engaged in the development of infantile kidneys.

We also observed that the cluster of genes participating in basement membrane integrity was highly expressed in infant kidneys compared to juvenile kidneys. Genes encoding extracellular matrix (ECM) components are well known to change their expression levels during kidney tubulogenesis and maturation [31]. Among them, collagen is a major component that provides structural integrity and its subtypes appear to have different expression levels during glomerular structural development in the kidney [31]. In our dataset, the members of the collagen IV family (*Col4a1*, *Col4a2*) showed two times higher expression in infant than juvenile kidneys (Fig. 3b). In concert with the overexpression of basement membrane composing protein, there was elevated expression of *Adamts2*, *Nid1*, *Serpinh1*, *and Wisp1* which participate in ECM synthesis [3, 25] (Fig. 3b). Genetic events regulating ECM deposition demonstrate that improvement of basement membrane integrity occurs in infants, which accounts for structural maturation after birth. In addition, genes that play roles in ureteric bud formation such as *Tbx10*, *Gpc3*, *Osr2*, and *Nid1* had higher expression in CON 1W than in CON 4W group. *Snail* is known to regulate mesenchymal to epithelial transformation [5]. Diminishing expression of *Snail* along with renal maturation in our dataset was coincident with previous work showing that *Snail* becomes inactive in the mature organ. Taken together, the results indicate that structural development of the kidney is not completed before birth, rather postnatal kidneys continue to grow during infancy and they undergo the processes necessary for mature structural integrity.

Contrary to the observation that structural morphogenesis was the most significant biological process in the transcriptome analysis of kidneys in CON 1W, upregulated genes in the CON 4W kidneys were associated with molecular transport, renal system development and function, and cellular function and maintenance (Fig. 3a). This indicates that functional maturation of the kidney was ongoing followed by morphological development. Genes involved in molecular transport associated with the renal system (*Kcnk5*, *Slc2a9*, *Slc6a9*, *Slc35a3*) were highly expressed with 1.6- to two-fold changes (Fig. 3b). The cytochrome P450 family, involved in bile acid metabolism in extrahepatic tissues, xenobiotics metabolism (*Cyp4b1*, *Ugt1a10*), and steroids and fatty acid metabolism (*Cyp2e1*, *Cyp4b1*, *Cyp7b1*, *Cyp4a12a*), was also upregulated during the juvenile period (Fig. 3b). These results indicate that the kidneys of juveniles are in the process of developing into functionally matured adult kidneys. We also checked whether aquaporin system that is critical for water transport in the kidney is in development and maturation. The increasing transcript levels of aquaporin family (*Aqp1*, *Aqp4*, and *Aqp8*; *p* < 0.01) with age support that functional maturation were taking place in the kidneys of infant and juvenile mice although the difference was slight (Fig. 3c).

### Dehydrated infant mice show altered Shh signaling pathway associated with impaired renal growth and development

**Fig. 4:**
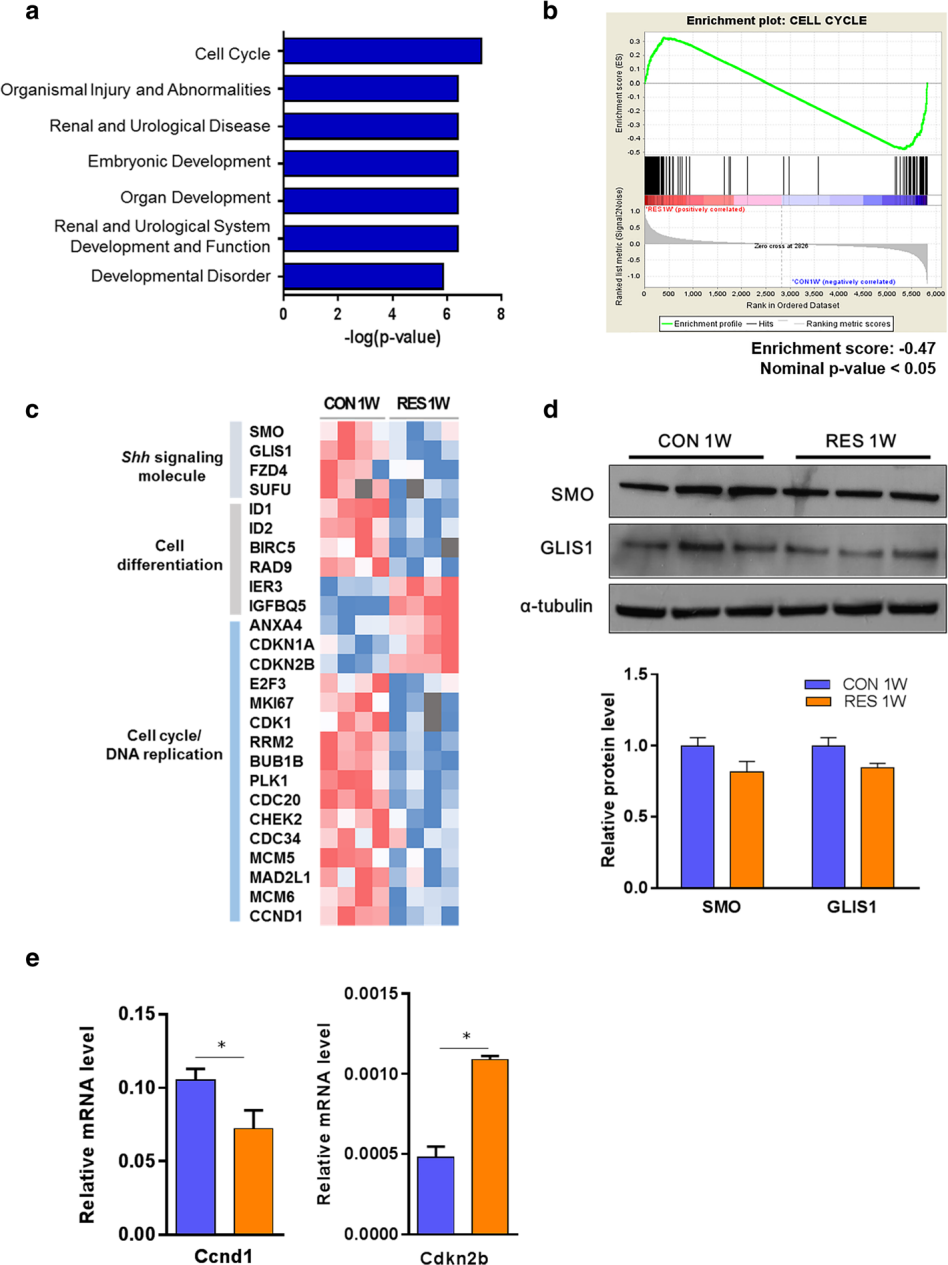
Dehydration interrupted renal morphogenesis by regulating Shh signaling in infant mice. **a** Top disease and functional categories of gene sets among differentially expressed genes in dehydrated mice during infancy. **b** Gene set enrichment analysis (GSEA) was used to evaluate the most significant functional category of differentially expressed genes by dehydration in infant mice and generate enrichment plot of cell cycle-related genes. **c** The heatmap shows downstream target genes of the SHH signaling pathway and indicates that cell cycle and cell differentiation regulators were distinctively regulated by dehydration. **d** Western blot assay was conducted for SHH signaling members. **e** The transcript level of its target molecules was validated with qPCR analysis. Data are expressed as mean ± SEM. Student’s *t* test; *p < 0.05 versus control group

Dehydration resulted in dramatic changes in kidney transcriptome profiles in infant mice. Each functional category of differentially expressed genes in the RES 1W group compared to CON 1W was tested for statistical significance. We found that the cell cycle, organ development, and renal system development were in the top seven significant categories (Fig. 4a). Gene set enrichment analysis (GSEA) confirmed that the cell cycle was the most enriched biological process regulated by hydration (Fig. 4b). These findings suggest that dehydration might have caused a deterioration in renal growth.

We further performed pathway analysis and found that one of the most crucial molecular pathways that were significantly regulated by dehydration during kidney organogenesis was the Sonic hedgehog (Shh) pathway. It is known to have a role in the expression of GLI transcription factors whose targets are cell cycle regulators [45]. Interestingly, a significant reduction in the *Shh* signaling pathway was observed following insufficient water intake. In concert with this, decreased expression of downstream target genes including cell cycle regulators (*Cdk1*, *Cdc20*, *Ccnd1*, *Plk1*, *Bub1b*, *Rrm2*, *Mcm5*, *Mcm6*) and cell differentiation-related molecules (*Id1*, *Id2*, *Rad9*, *Birc5*) was detected in the RES 1W group (Fig. 4c). Immunoblotting assays were conducted to validate the microarray analysis results for Shh proteins. SHH signaling molecules such as SMO and GLIS1 had a tendency to be reduced in the RES 1W group compared to the control group (*p* = 0.35) at the protein level (Fig. 4d). Target molecules in the SHH signaling, *Ccnd1* (1.7-fold decrease) and *Cdkn2b* (2.8-fold increase), were shown to be differentially regulated in the RES group by qPCR analyses (*p* < 0.05) (Fig. 4e).

### Dysregulation of transcriptional network associated with basement membrane integrity in juvenile mice by long-term effects of dehydration

**Fig. 5:**
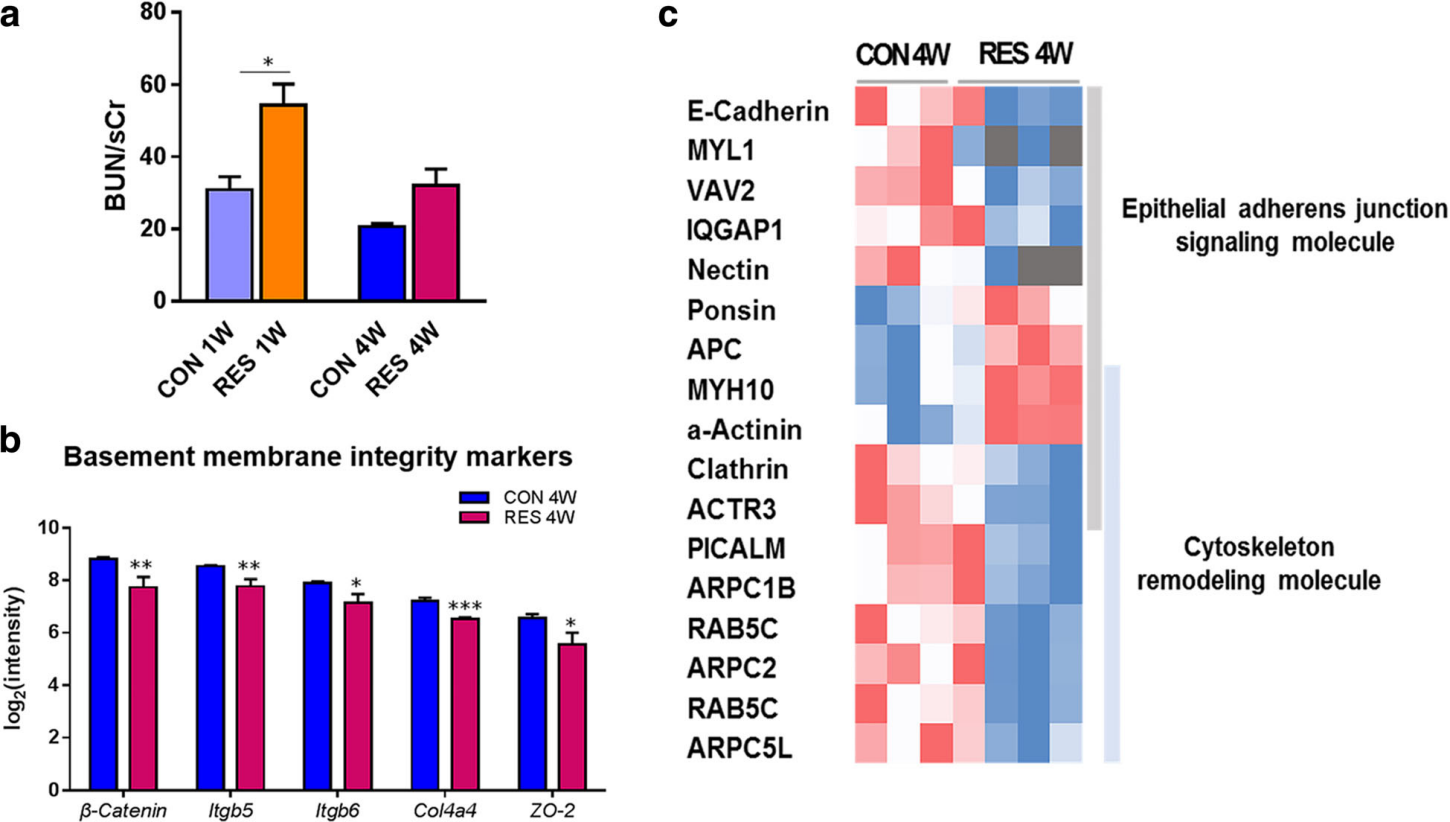
Disorders in basement membrane integrity by prolonged dehydration. **a** An increase in BUN/sCr level in the dehydration group compared to the control group. **b** Dysregulation of basement membrane components at the transcript level. **c** The heatmap shows the signaling molecules related to cell-cell junction and cytoskeleton rearrangement underlying altered membrane integrity. Data are expressed as mean ± SEM. Student’s *t* test; *p < 0.05, **p < 0.01, ***p < 0.001 versus control group

We observed a significant increase in blood urea nitrogen (BUN)/sCr in RES 1W (*p* < 0.05), and there was an increasing tendency in RES 4W (*p* = 0.08) in the age-matched comparison analysis after prolonged dehydration (Fig. 5a). To further investigate these results, we conducted comparison analysis of differentially expressed genes in the kidneys of RES 4W versus CON 4W. There were significant changes in basement membrane integrity markers. Renal epithelial basement membrane component (*Col4a4*; 1.6-fold decrease), integrins (*Itgb5* and *Itgb6*; 1.7-fold decreases), and molecules of cell adhesion including cell tight junctions or adherens junctions (β-catenin and ZO-2; twofold decreases) were reduced at the transcription level (Fig. 5b). These results may suggest that prolonged dehydration ultimately gave rise to breakdown of basement membrane assembly in the kidneys of juvenile mice, which can be secondary cause to functional disorders and renal pathophysiology.

In order to understand the implicit mechanism underlying the deterioration of membrane integrity, we confirmed the top significant pathways by canonical pathway analysis. We found that transcriptional events related to renal structural integrity were intrinsically altered via dysregulation of the epithelial adherens junction and its remodeling pathway (Additional file 3). Cadherin (*Cdh1*)-catenin (*Ctnnb1*) complex and nectin (*Pvrl1*) complex, which have roles in epithelial cell adhesion integrity and tissue homeostasis, were downregulated by sustained dehydration. Downstream molecules such as *Iqgap1*, *Actr3*, and *Vav2* were also decreased at the transcription level, indicating that alteration was observed in the network between cell-cell and cell-extracellular matrix contacts (Fig. 5c, Additional file 3). *Iqgap1* is a signaling molecule detected during the establishment of foot processes [27]. Especially, 3.4-fold increased expression of α-actinin-4, which is accompanied by foot progress effacement in damaged podocytes [32], was observed in the RES 4W group. In concert with depressed junction structural stability, many of the differentially regulated genes in the dehydration group were related to adherens junction recycling. The downstream effect of junction structure remodeling is implicated in clathrin-mediated endocytosis, which appeared to be suppressed in the dehydration group (Fig. 5c). Clathrin-mediated endocytosis is implicated in the cycling of cellular adhesion proteins [35]. Clathrin (*Cltc*), a mediator of vesicle formation during endocytosis of the cadherin complex, was decreased by 1.5-fold at the expression level in RES 4W. A gene encoding clathrin recruiting protein, *Picalm*, also showed decreased expression levels (1.7-fold change). *Rab5c*, with roles in vesicle docking and trafficking, had reduced transcription levels (1.6-fold change) along with depressed fusion of endocytic vesicles (Fig. 5c). From these results, we demonstrate that the dynamics of cytoskeleton and adherens junction signaling, which account for the membrane integrity, were dysregulated at the transcriptional level as a consequence of prolonged dehydration.

## Discussion

It was widely accepted that nephrogenesis completes during the embryonic period. However, recent studies unearthed new facts showing that later genetic events occur in postnatal renal growth. One study revealed that gene expression from E19.5 to P35 had a postnatal developmental pattern, showing a declining trend in cell differentiation and tissue development with aging in the mouse kidney [50]. In comparing the kidneys of adults to newborns, molecular transporters were found to be dominant in the transcriptome profiles [42]. Concurring with previous results, we identified that kidneys during infancy showed sequential processes indicating structural and functional maturation. Infantile kidneys, at 4 weeks old, were still differentiating and developed into functionally mature kidneys at 7 weeks old. This result provides critical evidence that postnatal care such as environmental and nutritional interventions is required for normal organ development since early renal dysfunction is implicated in various renal disorders in later life [13]. There is, however, a need for further studies to fully elucidate postnatal renal maturation using genetic perturbation animal models to elicit the importance of neonatal intervention for optimal growth.

Gene regulatory networks function in a coordinated fashion in kidney organogenesis to maintain kidney integrity [6]. It is, thus, emphasized that genetic developmental processes during the early postnatal life might be in charge of governing kidney health in later life. Moreover, despite the importance of adequate hydration in the maintenance of a healthier life, few studies have identified molecular mechanisms underlying the impacts of dehydration [17]. More than that, a fundamental methodological issue related to the experimental model of dehydration did not allow us to examine the true effects of dehydration. For instance, dehydration induced by acute water deprivation or heat and exercise stimulus could accompany or promote adverse physiological changes related to the dehydrating condition. Those also are incapable of generating consistent and controlled state of dehydration [28]. Here, we aimed to generate mild dehydration in animals, reproducing the condition of individuals that drink less water daily during the life cycle. With this approach, we were able to verify the molecular mechanisms underlying sustained moderate dehydration by profiling renal transcriptomes and anticipated its utility for the understanding of fundamental mechanisms underlying pediatric dehydration in the context of renal development.

We looked into the implications of dehydration throughout the early postnatal stage, from 4 to 7 weeks. Among corresponding mediators in kidney morphogenesis, the *Shh* signaling pathway and downstream target genes involved in the cell cycle and cell differentiation were downregulated as a consequence of dehydration. *Shh* is described as an important signaling molecule in renal patterning and the cell cycle during embryonic renal development [14]. Of its effectors, *Glis1* has a possible regulatory role in retaining normal morphological integrity in the kidney [20, 47]. Since the transcription level of *Glis1* was reduced and its target genes were changed in their expressions in the dehydration group, it is possible that genetic events associated with cell proliferation, differentiation, and death were implicated under improper hydration conditions.

As dehydration progressed throughout the developmental period, we found the transcriptional network involved in tissue integrity was deteriorated. Kidney integrity in structure and function is characterized by a series of successive modifications from neonates to aged mice [8, 37]. Sequential developmental processes in the kidneys correlate with numerous renal defects such as renal agenesis, hypoplasia, and polycystic kidney disease (PKD) [38]. In other words, normal renal organogenesis is necessary for mature functioning [8, 44, 48, 50] and diverse renal diseases are common consequences of abnormal kidney development [8, 38]. That is, it is conceivable that consecutive dehydration along with perturbation of morphogenesis influences sequential developmental steps including system functional maturation. One possible mechanism responsible for functional injury in the hyperosmolar state is demonstrated by the observation that continuous angiotensin II infusion into rats induced injuries in various cell types with worsened renal function [18]. In line with this report, kidney-specific cellular organization that is composed of networks between podocytes and the glomerular basement membrane might be damaged as the renin-angiotensin system was activated (data not shown) in the dehydration group. Genes associated with glomerular and tubular architecture, which includes basement membrane, slit diaphragm, and junction structure showed different mRNA expression patterns. In addition, many genes related to adherens junction-cytoskeleton dynamics were shown to be downregulated by dehydration. In fact, precise regulation of junctional adhesion molecules is a decisive element in the assembly of the actin cytoskeleton, ensuring tissue homeostasis and recovery from injuries [11]. These adhesion molecules have pivotal roles in renal epithelial cell dedifferentiation, proliferation, and migration. It is likely that the disruption in structural maturation during infancy resulted in defects in morphological integrity in terms of the loss of cell-cell and cell-membrane contacts in juvenile kidneys and failed adaptation to the loss of cells during regeneration [4]. Meanwhile, once renal cells are injured, loss of intact cells is directly linked to the loss of nephrons [32]. Due to degenerative changes in cellular integrity along with podocyte foot effacement, a limited number of intact nephrons are exposed to hyperfiltration. Therefore, overload of glomerular flow per unit of the kidney might result in glomeruli injuries and subsequent renal functional perturbation, leading to increased BUN/sCr ratio [10]. It is, therefore, conceivable that reduced membrane integrity impairs glomerular barrier function followed by improper tissue morphogenesis and homeostasis. However, it still needs histological and functional analysis to show direct evidence of physiological derangement or pathological changes of renal morphology in future studies.

Interestingly, prolonged dehydration during infancy resulted in reduced plasma creatinine levels. Based on present knowledge, reduction in the pool size or rate of creatinine metabolism, known as a “creatinine deficit,” may account for the reduction in plasma creatinine under pathophysiologic conditions [12, 19]. Otherwise, we assume that failure in toxic substance clearance by renal insufficiency resulted in hyper toxic loads, augmenting systemic disorders in animals. While plasma creatinine is one of the most frequently used markers for kidney disease, it could blunt the early diagnosis of pediatric renal diseases in cases where the clinical marker is arbitrarily estimated. We, hereby, suggest that plasma creatinine might underestimate the significance of renal disorders in pediatric dehydration. Thus, it is noted that different clinical markers are required for pediatric renal disorders. Meanwhile, at the onset of dehydration, dehydrated mice exhibited reduced diet consumption during the first few days (Additional file 4). Despite catching up to normal diet intake after the first week of the dehydration experiment, insufficient energy intake could be a cause of developmental disorders in the situation of impaired renal development in pediatric dehydration. Notwithstanding, it is noted that animals have different patterns of eating behavior with reduced appetite under high osmolar stimulus while they are subjected to strict control of osmolality [15]. Given the complexity of the physiological response, therefore, we suggest that changes in eating behavior are a validated consequence of physiological adaptation to water deprivation [15].

In this research, we demonstrate transcriptional events during renal development in infancy and show that the impacts of inadequate water intake in the early postnatal stage heavily rely on the impairment of normal renal maturation. This finding suggests that optimal nutritional intervention would be required for successful renal development.

## Conclusions

Altogether, this study gives us better insights into the effects of dehydration on functionally immature kidneys of infants and may provide possible markers for clinical applications in pediatric dehydration. In this respect, this study could be a cornerstone providing predictive biomarkers for future examination of known renal diseases and a broadened perspective of pediatric renal defects during development.

## Additional files

Additional file 1: Differentially expressed genes during normal renal development from infancy to juvenile period. Differentially expressed genes in a comparison analysis between CON 1W and CON 4W group, with statistical significant change (*p* < 0.05 and fold change >1.5). (XLSX 24 kb)
Additional file 2: Upstream regulators in infantile renal development. Upstream regulator analysis shows the top predicted regulators that connected to the downstream nodes. Activation z-score of each regulator is presented in brackets. Edges indicate predicted relationships, colored with yellow and blue when it led to activation and inhibition, respectively. (TIF 8929 kb)
Additional file 3: Dysregulation of transcriptional network associated with cell junction dynamics. (A) Top canonical pathways that were differentially regulated following dehydration in juvenile mice. (B) Suppression of adherens junction signaling accounts for altered cytoskeleton rearrangement, which caused alteration of glomerular barrier integrity. Genes in green and red color were down- and upregulated, respectively. (TIF 5873 kb)
Additional file 4: Diet intake during experimental period. Animals in the dehydration group exhibited reduced diet consumption during the first few days but caught up to normal diet intake after the first week of the dehydration experiment. Data are expressed as mean ± SEM. Student’s *t* test; **p* < 0.05 versus control group. (TIF 45 kb)

## Abbreviations

Avp: Arginine vasopressin
BUN: Blood urea nitrogen
ECM: Extracellular matrix
GO: Gene ontology
GSEA: Gene set enrichment analysis
PCA: Principal component analysis
PKD: Polycystic kidney disease
sCr: Serum creatinine
SHH: Sonic hedgehog
