# Supplementary figures and images for: Improper hydration induces global gene expression changes associated with renal development in infant mice
Source: Genes Nutr. 2016 Oct 20;11:28. doi: 10.1186/s12263-016-0544-0 (PMC5072351; doi:10.1186/s12263-016-0544-0)

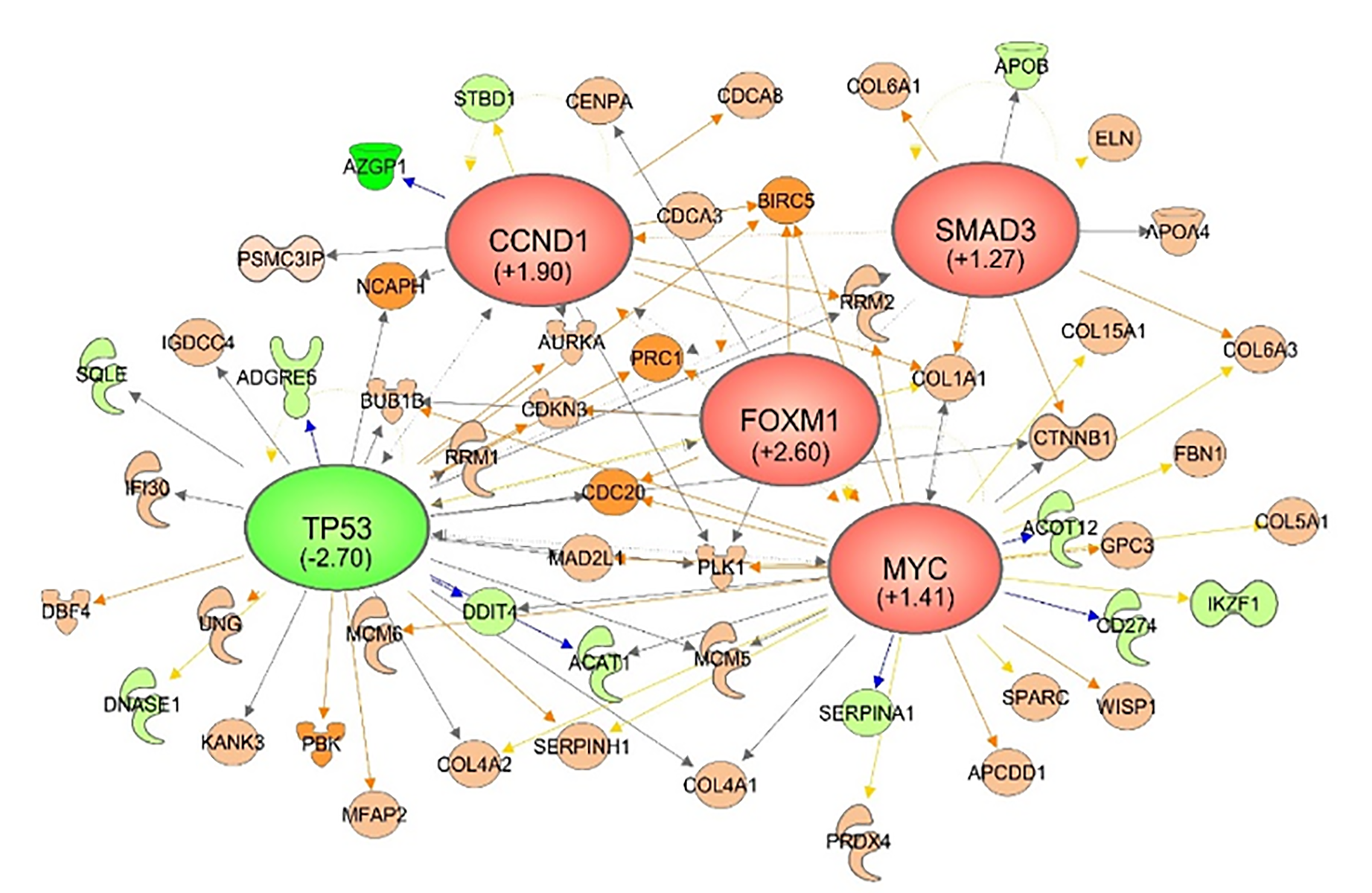

Supplement: Additional file 2: — Upstream regulators in infantile renal development. Upstream regulator analysis shows the top predicted regulators that connected to the downstream nodes. Activation z-score of each regulator is presented in brackets. Edges indicate predicted relationships, colored with yellow and blue when it led to activation and inhibition, respectively. (TIF 8929 kb) [file 12263_2016_544_MOESM2_ESM.tif]

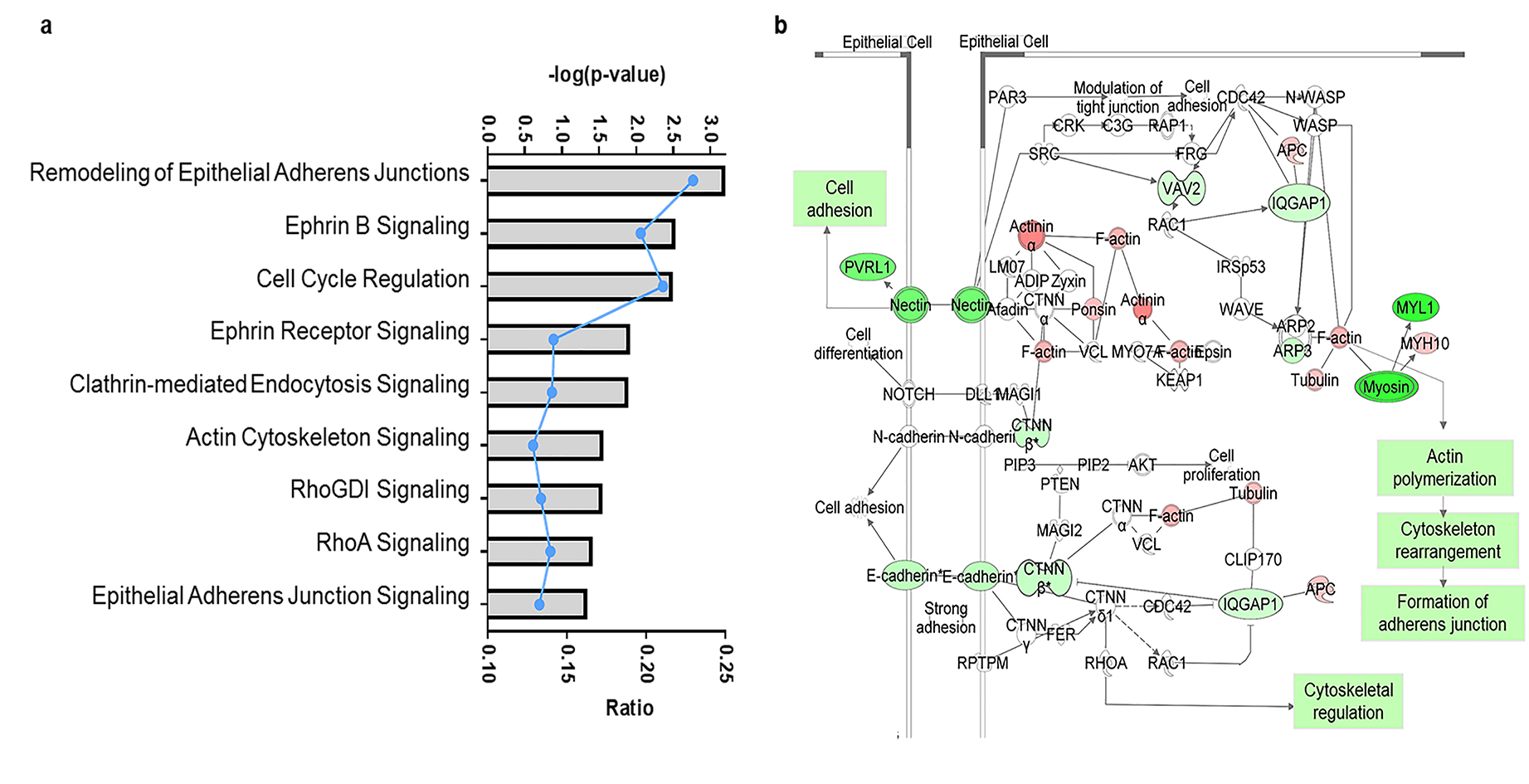

Supplement: Additional file 3: — Dysregulation of transcriptional network associated with cell junction dynamics. (A) Top canonical pathways that were differentially regulated following dehydration in juvenile mice. (B) Suppression of adherens junction signaling accounts for altered cytoskeleton rearrangement, which caused alteration of glomerular barrier integrity. Genes in green and red color were down- and upregulated, respectively. (TIF 5873 kb) [file 12263_2016_544_MOESM3_ESM.tif]

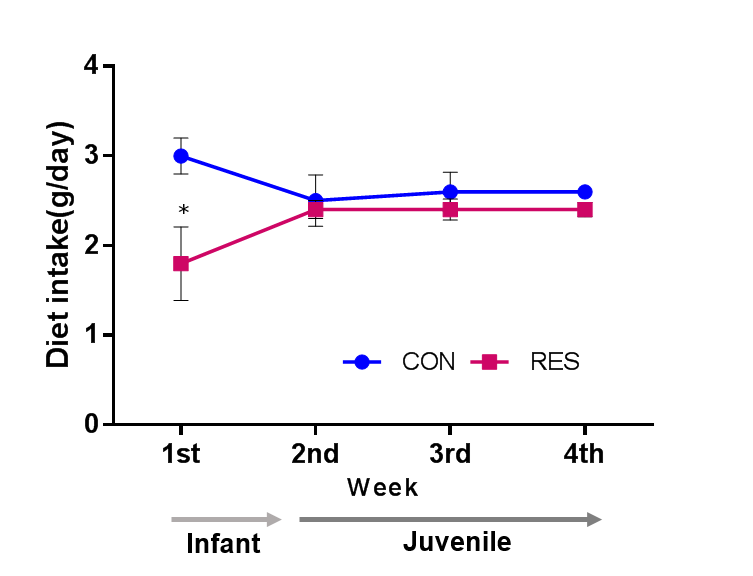

Supplement: Additional file 4: — Diet intake during experimental period. Animals in the dehydration group exhibited reduced diet consumption during the first few days but caught up to normal diet intake after the first week of the dehydration experiment. Data are expressed as mean ± SEM. Student’s t test; *p < 0.05 versus control group. (TIF 45 kb) [file 12263_2016_544_MOESM4_ESM.tif]
